# Comparative effects of combined omeprazole and dose-escalated striatin therapy versus monotherapy on VEGF, ITF, gastric ulcer area, epithelial thickness, and inflammatory response in rats with aspirin-induced gastric injury

**DOI:** 10.3389/fphar.2026.1816185

**Published:** 2026-06-10

**Authors:** Hery Djagat Purnomo, Meita Hendrianingtyas, Reza Rachman, Sarah Nadya Roosana, Didik Indiarso, Hesti Triwahyu Hutami, Cecilia Oktaria Permatadewi, Hermawan Istiadi, Pulong Wijang Pralampita, Dharminto, Raymond R. Tjandrawinata

**Affiliations:** 1 Division of Gastroentero-Hepatology, Department of Medicine, Faculty of Medicine, Universitas Diponegoro/Dr. Kariadi Central General Hospital, Semarang, Indonesia; 2 Department of Clinical Pathology, Faculty of Medicine, Universitas Diponegoro, Semarang, Indonesia; 3 Specialist Medical Education Program, Department of Medicine, Faculty of Medicine, Universitas Diponegoro/Dr. Kariadi Central General Hospital, Semarang, Indonesia; 4 Department of Anatomical Pathology, Faculty of Medicine, Universitas Diponegoro, Semarang, Indonesia; 5 Coordinator of Animal Laboratory, Faculty of Medicine, Universitas Diponegoro, Dr. Kariadi Central General Hospital, Semarang, Indonesia; 6 Department of Biostatistics and Population, Faculty of Public Health, Universitas Diponegoro, Semarang, Indonesia; 7 School of Bioscience, Technology and Innovation, Atma Jaya Catholic University of Indonesia, Jakarta, Indonesia

**Keywords:** *Channa striata*, epithelial integrity, gastric ulcer, gastritis, inflammatory response, intestinal trefoil factor, omeprazole, vascular endothelial growth factor

## Abstract

**Introduction:**

Aspirin-induced gastritis results from reduced prostaglandin synthesis and increased oxidative stress, compromising gastric mucosal integrity. While omeprazole effectively suppresses gastric acid secretion, it does not directly promote mucosal regeneration. Striatin, a bioactive fraction of *Channa striata*, has anti-inflammatory and regenerative properties and may enhance mucosal healing.

**Purpose:**

This study aimed to evaluate whether adjunctive striatin at escalating doses enhances the therapeutic effect of omeprazole in aspirin-induced gastric injury.

**Methods:**

A controlled experimental study was conducted in 30 male Wistar rats randomly assigned into six groups (n = 5 each): healthy control, negative control, omeprazole monotherapy (20 mg/kg BW), and three combination groups receiving omeprazole plus striatin (500, 1,000, and 1,500 mg/kg BW). Gastric injury was induced using aspirin. Vascular endothelial growth factor (VEGF; day 2) and intestinal trefoil factor (ITF; day 14) were measured as markers of angiogenesis and epithelial restitution, respectively. Histological and macroscopic assessments were performed at the end of treatment.

**Results and Discussion:**

VEGF levels did not significantly differ across groups. In contrast, ITF significantly increased in the highest-dose striatin group (1,500 mg/kg BW, *p* = 0.007), which also demonstrated improved epithelial thickness, reduced ulcer area, and lower inflammation scores. Lower doses did not produce significant changes in ITF levels.

**Conclusion:**

Adjunctive striatin, particularly at higher doses, enhances gastric mucosal healing primarily through epithelial restitution rather than angiogenesis. These findings suggest potential adjunctive benefits, although further studies are needed to establish optimal dosing and clinical relevance.

## 1 Introduction

Aspirin is a major cause of gastric mucosal injury because cyclooxygenase inhibition reduces prostaglandin-mediated epithelial and microvascular protection (Vane and Botting, 2003). The resulting ischemia, oxidative stress, and epithelial necrosis initiate leukocyte recruitment and cytokine release, amplifying mucosal damage. Gastric ulcer healing then progresses through tightly coordinated phases of epithelial regeneration, granulation tissue formation, angiogenesis, and matrix remodeling, culminating in scar restoration. These events are regulated by interconnected growth factor, cytokine, and transcription factor signaling networks (Vane and Botting, 2003; Treuting et al., 2018; Braga Emidio et al., 2020).

Taken together, current evidence suggests that effective gastric ulcer healing requires more than luminal acid suppression. Although proton pump inhibitors such as omeprazole remain central to the management of NSAID-related gastropathy, their therapeutic effect is primarily limited to reducing gastric acidity and stabilizing the intraluminal environment. Acid suppression alone does not directly activate epithelial restitution, growth factor signaling, or late-phase mucosal remodeling, processes that are critical for complete structural and functional recovery of the gastric barrier (Bjarnason et al., 2018). With increasing reports of long-term PPI-associated adverse effects, there is growing interest in therapeutic strategies that target endogenous regenerative pathways to achieve more complete mucosal recovery. Consequently, there is increasing interest in adjunctive strategies that can biologically complement acid suppression by actively engaging endogenous regenerative pathways.

Striatin, a standardized bioactive protein fraction derived from *Channa striata*, has emerged as a promising regenerative adjunct due to its documented anti-inflammatory, antioxidant, and wound-healing properties. Unlike conventional gastroprotective agents, striatin targets epithelial repair biology by modulating trefoil factor signaling and supporting cellular migration, barrier restitution, and tissue remodeling (Rahayu et al., 2016; Hapsari and Tjandrawinata, 2025; Yulizal et al., 2020). Importantly, gastric healing is a temporally structured process in which angiogenic mediators such as vascular endothelial growth factor (VEGF) dominate the early repair phase, whereas epithelial restitution markers such as intestinal trefoil factor (ITF) become more relevant during the later stages of mucosal recovery (Thim and May 2022). However, the extent to which a dose-escalated regenerative adjunct can differentially influence these temporally distinct healing pathways when combined with acid suppression remains insufficiently characterized.

Therefore, this study was designed to evaluate whether escalating doses of striatin, administered as an adjunct to omeprazole, can shift the gastric healing phenotype beyond acid suppression alone in an aspirin-induced gastric injury model. By integrating biochemical markers representing early angiogenic response (VEGF) and late epithelial restitution (ITF) with histopathological and macroscopic healing indices, this study aims to clarify not only whether combination therapy improves outcomes but also how it modulates the biological trajectory of gastric mucosal repair.

Although previous studies have demonstrated the gastroprotective effects of *Channa striata* and its derivatives as monotherapy, the potential benefit of combining striatin with proton pump inhibitors has not been well established. In particular, it remains unclear whether such combination therapy can enhance mucosal healing beyond acid suppression via distinct biological pathways, including angiogenesis and epithelial restitution. Therefore, this study specifically evaluates the adjunctive role of dose-escalated striatin combined with omeprazole using integrated biochemical and histopathological outcomes.

## 2 Materials and methods

### 2.1 Study design

This research used a controlled experimental laboratory design to evaluate whether combining omeprazole with graded doses of striatin provides greater mucosal protection and repair than omeprazole alone in aspirin-induced gastric injury. The research was conducted at the Laboratory for Animal Experimentation, Faculty of Medicine, Universitas Diponegoro, Semarang, Indonesia, using 30 male Wistar rats assigned to a pre-test–post-test control group model. Animals were randomly allocated into six groups: a healthy control group (K0), a negative control group (K−), a positive control group (K+) receiving omeprazole monotherapy (20 mg/kg BW), and three treatment groups (P1, P2, and P3) receiving a combination of omeprazole (20 mg/kg BW) with striatin at doses of 500, 1,000, and 1,500 mg/kg BW, respectively. Animals were randomly allocated using a computer-generated randomization sequence. All procedures were conducted under standardized conditions, and investigators performing the assessments were blinded to treatment allocation to minimize observational bias.

### 2.2 Animals and ethical approval

Male *Rattus norvegicus* Wistar rats (8–12 weeks old; 200–300 g) were used in this study. Animals were housed in a controlled environment maintained at 22 °C–24 °C with 70%–75% relative humidity and a 12-h light–dark cycle. Rats were accommodated in sanitized polypropylene group cages (50 × 40 × 20 cm; three rats per cage) with corn cob bedding and were provided standard BR-594 chow and water *ad libitum*. Animals were acclimatized for 7 days before experimentation to reduce physiological stress. All procedures for animal handling, housing, and welfare followed the ARRIVE 2.0 guidelines and international standards for laboratory animal care. Ethical approval was obtained from the Health Research Ethics Committee of the Faculty of Medicine, Universitas Diponegoro/RSUP Dr. Kariadi Semarang (Ethical Clearance No.: 110/EC/KEPK/FK-Undip/V/2025). Sample size was determined using power analysis to achieve sufficient statistical power while adhering to the principles of the 3Rs (replacement, reduction, and refinement).

### 2.3 Gastric injury induction and treatment procedures

Gastric injury was induced using oral aspirin, which induces mucosal erosions through prostaglandin inhibition and direct epithelial irritation. Prior to induction, rats were fasted for 24 h with free access to water. Aspirin was administered orally at a dose of 450 mg/kg body weight, prepared by suspending the powder in 0.5% CMC. The aspirin suspension was delivered once daily via intragastric gavage for 18 consecutive days to all rats except those in the healthy control group. Animals were observed throughout the induction period to verify the successful establishment of the gastric injury model before initiating treatment.

After aspirin induction, the animals proceeded to the treatment phase and received either omeprazole alone or omeprazole in combination with increasing doses of striatin. The negative control group received aspirin induction without therapeutic intervention, whereas the positive control group was administered omeprazole monotherapy at a dose of 20 mg/kg body weight. All treatments were delivered once daily via intragastric gavage for 14 days (day 26 to day 39) to ensure consistent and controlled dosing.

Omeprazole was prepared at a dose of 20 mg/kg BW by suspending the powdered formulation in 0.5% CMC, and the suspension was administered orally. Striatin, supplied by Dexa Laboratories of Biomolecular Sciences (DLBS; Bekasi, West Java, Indonesia), was obtained from *Channa striata* fillets through water-based extraction followed by fractional purification, concentration, and stabilization with sodium alginate, resulting in a standardized bioactive protein fraction produced under Good Manufacturing Practice conditions. Striatin was administered at doses of 500, 1,000, and 1,500 mg/kg BW, prepared in 0.5% CMC, and co-administered with omeprazole throughout the 14-day treatment period. All administrations were performed via oral gavage to maintain dosing precision and uniformity.

### 2.4 Blood and gastric tissue collection

Blood samples were collected from the retro-orbital vein. Before the intervention phase, baseline serum sampling was performed in all rats on day 22 for VEGF measurement and day 24 for ITF measurement. VEGF was re-measured on day 27 (early treatment phase; day 2 of intervention) to capture early angiogenic response, while ITF was re-measured on day 39 (end of treatment) to reflect late epithelial restitution. Approximately 100 µL of serum was collected for each assay at every time point. Gastric tissue was obtained from all groups on day 39, 24 h after the final intervention dose.

### 2.5 Biochemical analysis

Levels of VEGF and ITF were quantified by sandwich ELISA, with all samples analyzed in duplicate according to the manufacturer’s instructions. Blood was centrifuged at 1000 *g* to separate serum, which was then stored at −20 °C to −80 °C until analysis to prevent degradation from repeated freeze–thaw cycles. Calibrators were prepared through serial dilution to generate an eight-point standard curve. For each assay, 100 µL of standards or samples was added to the wells and incubated at 37 °C, followed by sequential incubation with detection reagents A and B, substrate addition, and termination of the reaction. Absorbance was measured at 450 nm using a microplate spectrophotometer.

### 2.6 Macroscopic evaluation of gastric ulcer area

**FIGURE 1:**
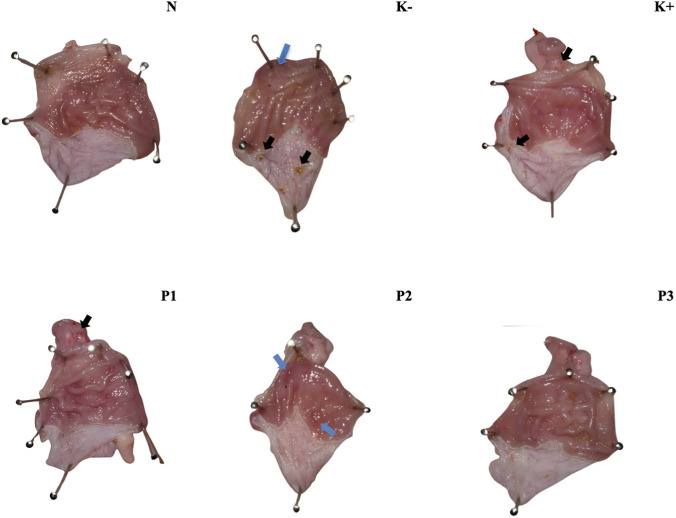
Macroscopic evaluation of gastric ulcers. Black arrows indicate ulceration; blue arrows indicate mucosal erythema.

Following euthanasia, stomachs were dissected, opened along the greater curvature, gently rinsed with saline, and positioned flat for macroscopic examination. Ulcerative lesions were identified as distinct areas of hemorrhage or necrosis. Digital images were obtained under standardized lighting conditions by examiners blinded to treatment allocation. Gastric ulcer severity was assessed using a standardized scoring scale based on lesion number and diameter: 0 = no lesion; 1 = edematous/erythematous mucosa; 2 = 1–5 small lesions; 3 = 5 small or 1 moderate lesion (3–4 mm); 4 = ≥2 moderate lesions or 1 large lesion (>4 mm); and 5 = perforated ulcer. Macroscopic evaluations were performed by an expert anatomical pathologist, with representative findings shown in Figure 1.

### 2.7 Histological assessment and epithelial thickness

**FIGURE 2:**
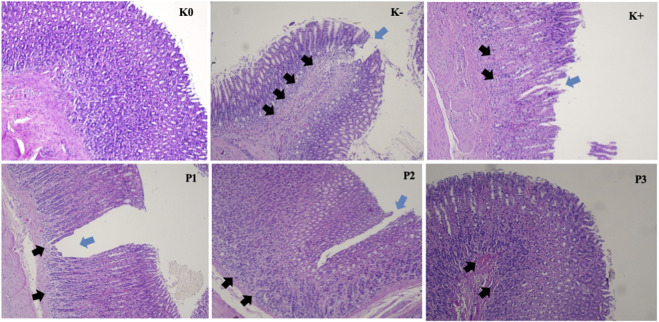
Gastric histopathological findings in each group (hematoxylin and eosin staining, ×100). Black arrows indicate inflammatory cell infiltration, and blue arrows indicate areas of gastric erosion.

Histopathological slide preparation was performed according to the standardized protocol established in the dose-finding phase. Gastric tissue samples were fixed in buffered formalin, embedded in paraffin, sectioned, and stained with hematoxylin and eosin. Histopathological parameters were assessed, including gastric epithelial thickness, which was measured microscopically using a calibrated ocular micrometer across five representative fields per specimen. Epithelial thickness was also evaluated categorically in comparison with the healthy control group (K0) and classified into four categories: 25%–50%, 50%–75%, 75%–100%, or 100% of normal mucosal thickness. All histopathological evaluations were conducted by an expert anatomical pathologist blinded to treatment allocation, as illustrated in Figure 2. However, because a semi-quantitative scoring system was used, observer bias cannot be entirely excluded.

### 2.8 Inflammation scoring

The inflammatory response was evaluated using a semi-quantitative scoring system based on the degree of inflammatory cell infiltration and the extent of mucosal disruption. Inflammation severity was classified into four categories: no infiltration, mild infiltration, moderate infiltration, and severe infiltration. Histopathological assessment was performed by an expert anatomical pathologist blinded to the treatment allocation to minimize assessment bias, as shown in Figure 2. The use of a semi-quantitative scoring system may introduce subjectivity despite blinding procedures.

### 2.9 Statistical analysis

Data were expressed as the mean ± standard deviation (SD) or median (Q1–Q3), as appropriate. Normality was assessed using the Shapiro–Wilk test. Pre- and post-intervention comparisons were analyzed using the paired t-test or Wilcoxon test, while between-group comparisons were analyzed using the independent t-test or Mann–Whitney test. For multiple group comparisons, one-way ANOVA with post-hoc testing was used for normally distributed data, and the Kruskal–Wallis test followed by the Mann–Whitney test was applied otherwise. Delta values were calculated to assess changes from baseline and were analyzed accordingly. Statistical significance was defined as *p* ≤ 0.05. No formal correction for multiple comparisons was applied; therefore, the results should be interpreted with caution due to the potential increased risk of type I error.

## 3 Results

### 3.1 Characteristics of experimental animals

A total of thirty male Wistar rats were enrolled and allocated into six groups after a seven-day acclimatization period under controlled housing conditions. Baseline characteristics, including body weight and general condition, were comparable across groups, and no clinically relevant differences were observed prior to treatment administration.

### 3.2 Vascular endothelial growth factor

Serum VEGF concentrations varied across individuals but showed comparable baseline levels among groups. Only the positive control group exhibited a significant within-group increase after treatment, while the remaining groups did not demonstrate significant changes. Between-group comparisons after treatment showed no statistically significant differences (*p* > 0.05). These findings indicate that neither omeprazole monotherapy nor its combination with striatin significantly altered VEGF levels under the current experimental conditions. The complete VEGF results are presented in Table 1.

**TABLE 1:** Statistical analysis of VEGF levels before and after treatment across groups.

Group	VEGF pre	VEGF post (minimum–maximum)	P	ΔVEGF
K0	7.53 (6.01–12.69)	4.50 (3.51–14.26)	0.500†	−4.02 (−4.65–8.25)
K–	11.65 (7.02–20.62)	4.26 (6.01–35.91)	0.274¶	0.00 (−3.70–24.26)
K+	4.50 (2.53–10.61)	13.21 (8.55–14.78)	0.013¶*	4.69 (2.60–10.22)
P1	9.06 (3.02–14.26)	10.09 (3.02–16.37)	0.601¶	2.11 (−7.59–11.76)
P2	8.55 (1.57–13.73)	6.51 (4.01–22.23)	0.828¶	0.00 (−7.22–12.14)
P3	8.04 (2.53–13.21)	15.31 (11.13–19.55)	0.057¶	7.27 (0.00–14.04)
p	0.187§	0.144‡	​	0.199‡

*significant (*p* < 0.05); † Wilcoxon; ¶ paired t-test; § one-way ANOVA; ‡ Kruskal–Wallis.

### 3.3 Intestinal trefoil factor

ITF concentrations increased following treatment, with a statistically significant increase observed only in the highest-dose combination group (1,500 mg/kg BW) compared to baseline (*p* = 0.007). Baseline ITF levels were comparable across groups, while no significant changes were observed in the lower-dose groups (500 and 1,000 mg/kg BW). Detailed values are presented in Table 2.

**TABLE 2:** Statistical analysis of ITF levels before and after treatment across groups.

Group	ITF pre	ITF post	p	ΔITF
K0	10,539.0 ± 860.4	10,308.0 ± 1371.6	0.521¶	−231.0 ± 734.3
K–	10,112.2 ± 595.1	10,454.8 ± 2052.0	0.766¶	342.6 ± 2,400.9
K+	8831.2 ± 1144.5	9430.2 ± 1070.5	0.174¶	599.0 ± 811.3
P1^a^	9165.4 ± 702.4	11,264.4 ± 2737.2	0.068¶	2,099.0 ± 1,892.6
P2	9293.6 ± 1674.5	9905.8 ± 994.7	0.448¶	612.2 ± 1,630.86
P3^a^ ^,^ ^b^ ^,^ ^c^ ^,^ ^d^	8363.0 ± 1407.8	12,191.4 ± 1696.6	0.008¶*	3,828.4 ± 1,740.6
P	0.055§	0.089§	​	0.007§*

*significant (*p* < 0.05); paired t-test; § one-way ANOVA.

K0, normal control; K−, negative control; K+, positive control (omeprazole 20 mg/kg BW); P1, combination of omeprazole and striatin (500 mg/kg BW); P2, combination of omeprazole and striatin (1,000 mg/kg BW); P3, combination of omeprazole and striatin (1,500 mg/kg BW).

^a^ *p* < 0.05 by the post-hoc Mann–Whitney test compared to the K0 group.

^b^ *p* < 0.05 by the post-hoc Mann–Whitney test compared to the K− group.

^c^ *p* < 0.05 by the post-hoc Mann–Whitney test compared to the K+ group.

^d^ *p* < 0.05 by the post-hoc Mann–Whitney test compared to the P2 group.

### 3.4 Epithelial thickness

Aspirin-induced injury resulted in reduced epithelial thickness in the negative control group. In contrast, increased epithelial thickness was observed in all treatment groups. The highest-dose combination group (1,500 mg/kg BW) demonstrated the greatest epithelial thickness values among the treatment groups. Statistical analysis showed significant differences among groups (*p* < 0.05). These findings are summarized in Table 3.

**TABLE 3:** Statistical analysis of epithelial thickness.

Group	Median (minimum–maximum)	*p*
K0	587.95 (580.97–637.32)	<0.001
K–^a^	189.30 (163.7–211.36)	​
K+^a^ ^,^ ^b^	381.51 (350.89–388.14)	​
P1^a^ ^,^ ^b^ ^,^ ^c^	487.61 (423.11–536.97)	​
P2^a^ ^,^ ^b^ ^,^ ^c^	529.32 (506.76–552.29)	​
P3^b^ ^,^ ^c^ ^,^ ^d^ ^,^ ^e^	603.28 (563.92–606.82)	​

K0, normal control; K−, negative control; K+, positive control (omeprazole 20 mg/kg BW); P1, combination of omeprazole and striatin (500 mg/kg BW); P2, combination of omeprazole and striatin (1,000 mg/kg BW); P3, combination of omeprazole and striatin (1,500 mg/kg BW).

^a^ *p* < 0.05 by the post-hoc Mann–Whitney test compared to the K0 group.

^b^ *p* < 0.05 by the post-hoc Mann–Whitney test compared to the K− group.

^c^ *p* < 0.05 by the post-hoc Mann–Whitney test compared to the K+ group.

^d^ *p* < 0.05 by the post-hoc Mann–Whitney test compared to the P1 group.

^e^ *p* < 0.05 by the post-hoc Mann–Whitney test compared to the P2 group.

### 3.5 Categorical distribution of epithelial thickness

When epithelial thickness was analyzed categorically, the healthy control group predominantly showed full-thickness mucosa, whereas the aspirin-induced group demonstrated substantial epithelial loss. The intermediate- and high-dose combination groups (1,000 and 1,500 mg/kg BW) showed a higher proportion of samples in the upper thickness categories. Overall differences among groups were statistically significant (*p* < 0.05), with several significant pairwise comparisons observed. The categorical distribution is shown in Table 4.

**TABLE 4:** Categorical distribution of epithelial thickness.

Group	100%	75%–100%	50%–75%	25%–50%	*p*
K0	5 (100%)	0	0	0	<0.001*
K–^a^	0	0	4 (80%)	1 (20%)	​
K+^a^	0	2 (40%)	3 (60%)	0	​
P1^a^ ^,^ ^b^	0	3 (60%)	2 (40%)	0	​
P2^a^ ^,^ ^b^ ^,^ ^c^	0	5 (100%)	0	0	​
P3^a^ ^,^ ^b^ ^,^ ^c^	0	5 (100%)	0	0	​

K0, normal control; K−, negative control; K+, positive control (omeprazole 20 mg/kg BW); P1, combination of omeprazole and striatin (500 mg/kg BW); P2, combination of omeprazole and striatin (1,000 mg/kg BW); P3, combination of omeprazole and striatin (1,500 mg/kg BW).

^a^ *p* < 0.05 by the post-hoc Mann–Whitney test compared to the K0 group.

^b^ *p* < 0.05 by the post-hoc Mann–Whitney test compared to the K− group.

^c^ *p* < 0.05 by the post-hoc Mann–Whitney test compared to the K+ group.

### 3.6 Inflammatory score of gastric mucosa

Inflammatory infiltration was absent in the healthy control group but present in the aspirin-induced group. Lower inflammation scores were observed in the treatment groups; however, no statistically significant differences were found between treatment groups. The overall comparison across groups was statistically significant (*p* < 0.05), primarily due to differences between the healthy control group and the other groups. Details are provided in Table 5.

**TABLE 5:** Inflammation degree across groups.

Group	No inflammation	Mild inflammation	*p*
K0	5 (100%)	0	<0.001*
K–^a^	0	5 (100%)	​
K+^a^	0	5 (100%)	​
P1^a^	0	5 (100%)	​
P2^a^	1 (20%)	4 (80%)	​
P3^a^	0	5 (100%)	​

K0, normal control; K−, negative control; K+, positive control (omeprazole 20 mg/kg BW); P1, combination of omeprazole and striatin (500 mg/kg BW); P2, combination of omeprazole and striatin (1,000 mg/kg BW); P3, combination of omeprazole and striatin (1,500 mg/kg BW); a *p* < 0.05 by the post-hoc Mann–Whitney test compared to the K0 group.

### 3.7 Gastric ulcer area

Macroscopic evaluation showed no ulceration in the healthy control group, whereas the aspirin-induced group exhibited evident gastric lesions. Reduced ulceration was observed in the treatment groups. No macroscopic lesions were detected in the highest-dose striatin combination group (1,500 mg/kg BW). Statistical analysis demonstrated significant overall differences among groups (*p* < 0.05), with multiple significant pairwise comparisons, particularly between the aspirin-induced group and all treatment groups. Full ulcer grading appears in Table 6.

**TABLE 6:** Ulcer area distribution across groups.

Group	No lesion	Edema–erythema	1–5 small lesions	≥5 small or 1 medium	≥2 medium or 1 large	*p*
K0	5 (100%)	0	0	0	0	<0.001*
K–^a^	0	0	0	2 (40%)	3 (60%)	​
K+^a^ ^,^ ^b^	0	0	3 (60%)	2 (40%)	0	​
P1^a^ ^,^ ^b^ ^,^ ^c^	0	3 (60%)	2 (40%)	0	0	​
P2^a^ ^,^ ^b^ ^,^ ^c^	0	5 (100%)	0	0	0	​
P3^b^ ^,^ ^c^ ^,^ ^d^ ^,^ ^e^	5 (100%)	0	0	0	0	​

K0, normal control; K−, negative control; K+, positive control (omeprazole 20 mg/kg BW); P1, combination of omeprazole and striatin (500 mg/kg BW); P2, combination of omeprazole and striatin (1,000 mg/kg BW); P3, combination of omeprazole and striatin (1,500 mg/kg BW).

^a^ *p* < 0.05 by the post-hoc Mann–Whitney test compared to the K0 group.

^b^ *p* < 0.05 by the post-hoc Mann–Whitney test compared to the K− group.

^c^ *p* < 0.05 by the post-hoc Mann–Whitney test compared to the K+ group.

^d^ *p* < 0.05 by the post-hoc Mann–Whitney test compared to the P1 group.

^e^ *p* < 0.05 by the post-hoc Mann–Whitney test compared to the P2 group.

## 4 Discussion

### 4.1 Interpretation of findings

VEGF analysis revealed a significant increase only in the omeprazole monotherapy group (K+), whereas the combination groups showed no substantial change and delta VEGF values remained non-significant. This pattern is consistent with the well-characterized temporal kinetics of VEGF, an angiogenic mediator that is rapidly induced via hypoxia-driven HIF-1α signaling immediately following mucosal injury. Because serum VEGF was measured on day 2 of treatment, the sampling window likely captured a post-peak intermediate phase of the angiogenic response. VEGF expression typically reaches its maximal elevation within the first 6–48 h after tissue insult; moreover, cessation of aspirin reduces the hypoxic stimulus for further VEGF transcription, and early luminal stabilization from omeprazole or striatin may have already diminished mucosal stress (Braga Emidio et al., 2020; Rahayu et al., 2016).

ITF analysis demonstrated a significant increase only in the highest-dose combination group (P3; 1,500 mg/kg BW). This suggests a potential threshold effect rather than a clear dose–response relationship. Given that ITF was measured on day 14, corresponding to the later phase of mucosal healing, this finding supports its role in epithelial restitution. However, the absence of significant changes in the lower-dose groups indicates that higher doses may be required to elicit measurable biological effects (Braga Emidio et al., 2020; Rahayu et al., 2016).

Integration of biomarker data with histopathological findings suggests that the highest-dose combination group (P3; 1,500 mg/kg BW) was associated with greater epithelial thickness, smaller ulcer areas, and lower inflammatory infiltration than the other groups. These findings are consistent with a more advanced stage of mucosal recovery; however, a direct causal relationship between biomarker changes and histological outcomes cannot be established (Hapsari and Tjandrawinata, 2025; Ali Khan et al., 2014).

### 4.2 Comparison with previous studies

Aspirin-induced ulcers are typically marked by epithelial loss, increased neutrophil infiltration, and delayed mucosal repair due to the suppression of protective prostaglandins and disruption of growth factor pathways (Bjarnason et al., 2018; Rahayu et al., 2016). Prior studies have shown that PPI therapy promotes ulcer healing but does not fully restore mucosal integrity, particularly when oxidative stress and cytokine imbalance persist (Hapsari and Tjandrawinata, 2025). The present findings are generally consistent with previous studies demonstrating that *Channa striata* extracts reduce inflammatory infiltration and support mucosal repair (Hapsari and Tjandrawinata, 2025; Yulizal et al., 2020; Mabrok and Mohamed, 2019). Similar patterns of epithelial restoration have been reported in experimental ulcer models, supporting the consistency of these findings with earlier evidence (Yibirin et al., 2021; Hong et al., 2020). The concurrent increase in ITF levels observed in our study mirrors reports that trefoil peptides increase after mucosal injury and contribute to restitution (Hoffmann, 2021).

### 4.3 Biological mechanisms

Aspirin-induced gastric injury begins with COX-1 and COX-2 inhibition, which suppresses prostaglandin synthesis and compromises the mucus–bicarbonate barrier, thereby increasing the mucosa’s vulnerability to luminal acid. Concurrently, aspirin provokes vascular and microvascular injury, leading to mucosal ischemia, hypoxia, oxidative stress, and subsequent epithelial necrosis. Necrotic tissue and leukotriene B serve as chemoattractants for leukocytes and macrophages, which amplify the inflammatory cascade by releasing cytokines such as TNF-α, IL-1α, and IL-1β. These mediators further activate local fibroblasts, endothelial cells, and epithelial cells, perpetuating tissue injury and delaying mucosal repair (Azemi et al., 2018).

Trefoil factor family (TFF), particularly ITF, plays a central role in gastric mucosal repair by promoting epithelial survival and rapid restitution through activation of ERK/MAPK, PI3K/AKT, PLC/PKC, β-catenin, and cross-talk with EGF pathways. ITF is the most strongly induced peptide following mucosal injury, as demonstrated in both experimental and human ulcer tissues, reflecting its key contribution to epithelial protection and regeneration. ITF also modulates the immune response by shifting the mucosal environment toward a Th2-dominant profile, thereby reducing pro-inflammatory Th1 activity and enhancing cytoprotective cytokines such as IL-4 and IL-13. Collectively, these actions accelerate epithelial restitution, reinforce the mucus barrier, stabilize tight junctions, and increase resistance to oxidative and chemical injury. Notably, TFF upregulation can occur earlier than EGF or TGF-α, indicating its potential role as an initiator of the healing cascade (Braga Emidio et al., 2020; Hoffmann, 2021; Tarnawski and Ahluwalia, 2021).

VEGF is a central regulator of angiogenesis and an endothelial-specific mitogen that exerts its effects through VEGF-R1 and VEGF-R2, thereby promoting endothelial proliferation, migration, and microvascular tube formation. Its expression is strongly induced by hypoxia via HIF-1 activation and further stimulated by mediators such as PDGF, TGF-β1, bFGF, nitric oxide, and prostaglandins. Experimental studies consistently demonstrate the essential role of VEGF in gastric ulcer healing, where exogenous VEGF or angiogenic co-factors (e.g., Ang1, bFGF, and PDGF) markedly accelerate granulation tissue formation, neovascularization, and restoration of glandular architecture. Conversely, blocking VEGF signaling impairs these processes, highlighting VEGF as a key determinant of effective mucosal repair (Braga Emidio et al., 2020; Tarnawski and Ahluwalia, 2021; Kobayashi et al., 2010).

Complementary mechanisms of action targeting multiple aspects of gastric mucosal repair may explain the observed improvements in the combination groups. Omeprazole reduces luminal acid exposure and thereby facilitates epithelial migration, stabilizes the healing environment, and protects regenerating glands from further injury (Lien et al., 2022). Striatin, in contrast, supplies a nutrient-dense biochemical substrate that directly supports mucosal regeneration. Its high content of essential amino acids, unsaturated fatty acids, vitamins, and minerals enhances epithelial restitution and reinforces mucosal barrier integrity. Unsaturated fatty acids, particularly oleic, linoleic, and palmitic acids, integrate into phospholipid membranes, improving membrane fluidity, structural stability, and resistance to oxidative injury. This membrane-restorative effect enhances epithelial cell viability, promotes migratory capacity, and optimizes the functional recovery of the mucosal surface. Concurrently, amino acids such as arginine, glutamine, proline, glycine, and cysteine contribute to collagen synthesis, fibroblast proliferation, antioxidant defense, and albumin restoration, collectively strengthening the mucosa’s reparative potential (Rahayu et al., 2016). Striatin contains copper, which contributes to increased VEGF expression within the wound environment. Arginine, which is oxidized to citrulline, serves as a cofactor in the conversion of eNOS to nitric oxide (NO). NO exerts protective effects by reducing platelet aggregation and promoting vasodilation, blood flow enhancement, and regulation of vascular tone. These mechanisms collectively improve tissue perfusion and oxygenation (Tarnawski and Ahluwalia, 2021; Cyr et al., 2020).

These biochemical mechanisms may help explain the observed biomarker patterns, although they were not directly assessed in this study. ITF increased significantly only in the highest-dose striatin group (P3), consistent with the central role of ITF in epithelial restitution, mucus barrier reinforcement, and tight junction stabilization. The cysteine-rich composition of striatin likely augments ITF activity by supporting its structurally conserved disulfide-stabilized trefoil domains. In contrast, VEGF did not increase significantly in the combination groups, a finding consistent with the sampling time on day 2—after the typical 6–24 h peak of hypoxia-induced VEGF expression—and does not exclude localized angiogenic effects, particularly given the presence of arginine, a precursor for nitric oxide–mediated endothelial activation (Hoffmann, 2021; Tarnawski and Ahluwalia, 2021).

The combination therapy may produce complementary effects by targeting distinct mechanisms of gastric repair. Omeprazole reduces acid exposure, thereby facilitating epithelial migration and protecting regenerating glands (Lien et al., 2022). Striatin, derived from *Channa striata*, contains peptides and amino acids with antioxidant and immunomodulatory properties that support fibroblast activity, angiogenesis, and epithelial proliferation (Bjarnason et al., 2018; Ruiz-Hurtado et al., 2021). These actions may support microvascular perfusion and epithelial repair, although a significant increase in VEGF was not observed in this study. Trefoil factor peptides also play a central role in mucosal restitution, and the observed increase in ITF is biologically consistent with improved epithelial migration and barrier restoration (Braga Emidio et al., 2020). By simultaneously addressing acid suppression, inflammation control, and regeneration pathways, the combination therapy provides a possible mechanistic rationale for the observed healing effects.

### 4.4 Clinical implications

Although this experimental study was conducted in an animal model, the findings may provide preliminary insights with potential clinical relevance. NSAID-related gastropathy remains a significant clinical burden, particularly in patients with chronic analgesic use or impaired mucosal defense (Braga Emidio et al., 2020). Standard prophylaxis with proton pump inhibitors reduces the risk of ulceration but does not consistently prevent mucosal injury or fully attenuate inflammatory responses. The present findings suggest that the addition of a bioactive regenerative agent such as striatin may serve as a potential adjunctive therapy, particularly in refractory cases or in patients with impaired mucosal healing. Striatin contains bioactive components that may support epithelial restitution and modulate inflammatory processes; however, its effects on angiogenesis were not clearly demonstrated in this study. Nevertheless, translation into clinical practice requires cautious interpretation. The doses used in this experimental model may not directly correspond to clinically feasible human doses, and further studies are needed to determine optimal dosing, safety, and efficacy. Well-designed clinical trials will be essential before considering its routine clinical application (Rahayu et al., 2016; Ali Khan et al., 2014).

### 4.5 Study limitations

This study has several limitations that should be considered. First, only short-term outcomes were evaluated, which limits the understanding of long-term mucosal remodeling and recurrence prevention. Second, molecular analyses were limited to VEGF and ITF, while other relevant mediators involved in ulcer healing, including EGF, PGE2, TGF-β, TFF1, TFF2, PDGF, nitric oxide, and COX-derived factors, were not assessed due to scope constraints. Third, striatin doses were selected based on previous experimental studies rather than pharmacokinetic optimization, and the bioavailability of its peptide components in gastric tissue remains incompletely characterized. Fourth, histological scoring was semi-quantitative and may not fully capture subtle differences in cellular regeneration. In addition, the relatively small sample size (n = 5 per group) may limit statistical power and increase the risk of type II error. Furthermore, multiple statistical comparisons were performed without adjustment, which may increase the risk of type I error. Finally, findings from rodent models may not fully extrapolate to human gastric physiology and therefore should be interpreted with caution.

### 4.6 Future directions

Future studies should explore a broader range of molecular markers involved in gastric mucosal repair to better define the underlying mechanisms of striatin. Mechanistic investigations focusing on epithelial restitution, angiogenesis, inflammatory modulation, and barrier integrity would help clarify its biological effects. Longitudinal studies are needed to evaluate sustained mucosal healing and recurrence following injury. In addition, further research should determine optimal dosing strategies and assess whether a threshold effect exists. Pharmacokinetic and bioavailability studies will also be important to better understand tissue distribution and therapeutic potential. Comparative studies with established gastroprotective agents may help define the relative efficacy of striatin. Ultimately, well-designed clinical trials will be required to evaluate its safety and effectiveness in human NSAID-related gastric injury.

## 5 Conclusion

In conclusion, the combination of omeprazole and striatin demonstrated improved gastric mucosal healing in an aspirin-induced rat model, as reflected by increased epithelial thickness, reduced ulcer area, and modulation of ITF levels. A significant increase in ITF was observed only at the highest striatin dose, suggesting a potential threshold effect. In contrast, VEGF levels did not differ significantly among groups, possibly due to the timing of measurement (Braga Emidio et al., 2020; Bjarnason et al., 2018; Rahayu et al., 2016).

These findings suggest that striatin may have potential as an adjunctive therapy by supporting mucosal repair processes; however, the underlying mechanisms were not directly established. Given the limitations of this study, including a small sample size and the use of an animal model, further research is required to confirm these findings and determine their clinical applicability.

## Data Availability

The raw data supporting the conclusions of this article will be made available by the authors, without undue reservation.
